# Acute hepatitis C infection among adults with HIV in the Netherlands between 2003 and 2016: a capture–recapture analysis for the 2013 to 2016 period

**DOI:** 10.2807/1560-7917.ES.2020.25.7.1900450

**Published:** 2020-02-20

**Authors:** T. Sonia Boender, Eline Op de Coul, Joop Arends, Maria Prins, Marc van der Valk, Jan T.M. van der Meer, Birgit van Benthem, Peter Reiss, Colette Smit

**Affiliations:** 1 Stichting HIV Monitoring, Amsterdam, the Netherlands; 2 National Institute for Public Health and the Environment (RIVM); Centre for Infectious Disease Control, Epidemiology and Surveillance, Bilthoven, the Netherlands; 3 Department of Internal Medicine, Division of Infectious Diseases, UMCU University Medical Center Utrecht, Utrecht University, Utrecht, the Netherlands; 4 Public Health Service of Amsterdam, Department of Infectious Diseases, Research and Prevention, Amsterdam, the Netherlands; 5 Department of Internal Medicine and Division of Infectious Diseases, Amsterdam University Medical Centers, Amsterdam Infection and Immunity Institute, University of Amsterdam, Amsterdam, the Netherlands; 6 Department of Global Health, Amsterdam University Medical Centers, University of Amsterdam, and Amsterdam Institute for Global Health and Development, Amsterdam, the Netherlands

**Keywords:** HIV Infections, Hepatitis C, Population Surveillance, Disease Notification, Registries, Netherlands

## Abstract

**Background:**

With regards to the global strategy towards eliminating viral hepatitis, reliable surveillance systems are essential to assess the national response for eliminating hepatitis C virus (HCV).

**Aim:**

We aimed to assess the completeness of the two national registries with data on acute HCV infection in people with HIV, and estimated the number of acute HCV infections among adults (aged ≥ 18 years) with HIV in the Netherlands.

**Methods:**

In this observational study, cases of HCV infection and reinfection among adults with a positive or unknown HIV-serostatus were identified from 2003 to 2016 in two national registries: the ATHENA cohort and the National Registry for Notifiable Diseases. For 2013–2016, cases were linked, and two-way capture–recapture analysis was carried out.

**Results:**

During 2013–2016, there were an estimated 282 (95% confidence interval (CI): 264–301) acute HCV infections among adults with HIV. The addition of cases with an unknown HIV-serostatus increased the matches (from n = 107 to n = 129), and subsequently increased the estimated total: 330 (95%CI: 309–351). Under-reporting was estimated at 14–20%.

**Conclusion:**

Under-reporting of acute HCV infection among people with HIV could partially be explained by an unknown HIV-serostatus, or by differences in HCV stage (acute or chronic) at first diagnosis. Surveillance data should ideally include both acute and chronic HCV infections, and enable to distinguish these as well as initial- and re-infections. National surveillance of acute HCV can be improved by increased notification of infections.

## Introduction

In 2016, the World Health Organization (WHO) set global targets to eliminate viral hepatitis as a major public health threat by 2030 [1]. In the European region, a specific action plan for the health response to viral hepatitis was put in place [2] and the global vision to eliminate viral hepatitis was translated into national strategies. In the Netherlands, all stakeholders in research, treatment, prevention and control of hepatitis B virus (HBV) and hepatitis C virus (HCV) jointly formulated a national hepatitis plan for action [3], which includes a call for improved HCV surveillance and monitoring.

HCV infections are generally uncommon in the Netherlands, with a chronic HCV prevalence of < 0.2% in 2016 [4]. However, an increase in the number of acute HCV infections and reinfections among men who have sex with men (MSM) who are human immunodeficiency virus (HIV)-positive has been reported since early 2000 [5-7]. Detection, diagnosis and registration of acute HCV infections are crucial to measure trends in the epidemic and to plan appropriate public health and clinical interventions, such as prevention programmes for those at risk, targeted testing, increasing treatment uptake, and contact tracing to reduce subsequent transmission. In addition, treating chronic HCV infections with direct acting antivirals (DAAs) is expected to strongly reduce, but not eliminate, the HCV epidemic among HIV-positive MSM [8,9]. Despite high DAA uptake in HIV/HCV co-infected people in the Netherlands [10], ongoing HCV transmission and reinfection continue to occur.

In the Netherlands, the Dutch HIV Monitoring Foundation (Stichting HIV Monitoring; SHM) [11] registers acute HCV infection among people with HIV since 2000, and the National Registry for Notifiable Diseases at the National Institute for Public Health and the Environment (Rijksinstituut voor Volksgezondheid en Milieu; RIVM) [7] registers acute HCV infections among people irrespective of HIV-serostatus since 2003. While the RIVM and SHM have reported similar trends of acute HCV infection over the years, consistently more cases were registered among HIV-positive MSM by the SHM, compared with the RIVM [7,12].

Reliable surveillance systems, monitoring and evaluation are essential to assess the national HCV response, in the context of the global strategy towards eliminating viral hepatitis [1,3]. In the absence of a national HCV registry, registry linkage studies are needed to assess and contextualise the available data on HCV infections, and to monitor progress towards HCV elimination. The question arises whether SHM and the RIVM register the same cases of acute HCV infection among people with HIV in the Netherlands, and whether cases are missed by either one of both registries. The aim of this study is to assess the completeness of these two national registries. In addition, we estimate the number of acute HCV infections among adults (≥ 18 years old) living with HIV in the Netherlands, by means of a capture–recapture analysis.

## Methods

Data were obtained from two national registries: the ATHENA national observational HIV cohort at SHM and the National Registry for Notifiable Diseases at the RIVM. We identified cases of acute HCV infection among adults (aged ≥ 18 years at HCV diagnosis) in the period of 2003–2016 in both registries. Both cases of a primary episode of acute HCV infection and reinfection were included; reinfections were treated as independent cases.

After describing the number of acute HCV infections among adults in both registries for the period of 2003–2016, we assessed the degree of overlap between the two registries for the period of 2013–2016 through case-linkage. By means of the capture–recapture method, we assessed the level of completeness of these two national registries, and estimated the number of acute HCV infections among adults living with HIV in the Netherlands.

### Data sources

#### ATHENA cohort at the Dutch HIV Monitoring Foundation

Since 2000, SHM has managed the ATHENA cohort and is responsible for registering all HIV-positive people in care at the 26 HIV-treatment centres in the Netherlands. At entry in HIV care, people are informed of the cohort and the purpose of data collection by their treating physician; participant consent follows an opt-out procedure (2% opt-out). After linkage to HIV care and registration at SHM, people are enrolled in the ATHENA cohort, which systematically collects demographic and clinical data through extraction from medical records, including information on HCV co-infection. The cohort does not schedule study visits: follow up of participants, and thereby the availability of data, depends on the frequency of clinical visits. Details about enrolment, data collection and follow up are described in the ATHENA cohort profile [11].

Of note, HIV is not a notifiable disease in the Netherlands. However, all people who attend an HIV-treatment centre are registered by SHM, and pseudonymised data on new cases of HIV infection are reported to the Dutch Centre for Infectious Disease Control (Centrum voor Infectieziektebestrijding; CIb) of the RIVM, for (inter)national HIV surveillance. Acute HCV infections are notifiable in the Netherlands. Acute HCV notification is the responsibility of the treating physician, as well as the diagnostic laboratory, as further described in this report. The SHM reports every year, in an aggregated manner, the epidemiology of HIV, including hepatitis C co-infections, among the ATHENA participants in an annual HIV monitoring report.

All cases of HCV infection were identified in the ATHENA cohort for the period of 2003 to 2016, based on the data as at May 2017 and also taking into account a retrospective update of the entries for that period in January 2018. Diagnosis of HCV infection was based on available laboratory results of HCV-RNA and anti-HCV IgG [5]. Our definition of acute HCV infection corresponds to the European Union (EU) case definition and the staging of cases (acute versus chronic) as performed by the European Centre for Disease Prevention and Control (ECDC) [13] and the European AIDS Treatment Network (NEAT) preferred criteria (Grade A, Level II) [14,15], and was defined as a positive anti-HCV IgG with a documented negative anti-HCV IgG within the previous 12 months, or a detectable HCV-RNA in the presence of either a documented negative HCV-RNA test or a documented anti-HCV IgG seroconversion within the previous 12 months. Additionally, cases of acute HCV reinfection were identified following sustained virological response, spontaneous clearance, or genotype switch.

Moreover, cases of chronic HCV infection were defined as HCV-RNA positivity for > 6 months. In case of an undetermined stage of HCV infection (i.e. acute or chronic), stage of infection was verified with the HIV-treating physician. Alternatively, if the stage of HCV infection remained unresolved, cases of HCV infection were considered in a separate category called ‘other cases of HCV infection’ for the purpose of this study. We defined the timing of HCV infection based on the date of the first laboratory diagnosis (RNA or antibody).

#### National registry for notifiable diseases at the National Institute for Public Health and the Environment

Since 1998, as a legal obligation enforced by the Dutch public health act (Wet publieke gezondheid) [16], both treating physicians and diagnostic laboratories must report cases of HCV infection, together with demographic and epidemiological information, to the local Public Health Service (Geneeskundige Gezondheidsdienst, GGD). The GGD subsequently reports to the National Registry for Notifiable Diseases at the RIVM, using the web-based application OSIRIS [17]. As of 1 October 2003, only acute HCV infections need to be reported to the RIVM. The RIVM collects additional information on the HIV-serostatus of people with an acute HCV infection, and whether the HCV infection is a primary infection or reinfection, since 1 January 2013. HIV infection is not a notifiable disease in the Netherlands; however, all people who attend an HIV-treatment centre are registered by SHM, and a subset of pseudonomised data from these individuals is shared with the CIb of the RIVM; also described in this report under ‘ATHENA cohort at the Dutch HIV Monitoring Foundation’.

Cases of acute HCV infection were identified in the National Registry for Notifiable Diseases at the RIVM for the period of 2003 to 2016, based on the database as at 27 February 2017.

The RIVM case definition corresponds to the SHM case definition (i.e. EU/ECDC [13] and the NEAT preferred criteria (Grade A, Level II)) and, in addition, includes diagnoses based on a positive HCV-RNA associated with an elevated alanine aminotransferase according to the NEAT alternative criteria (Grade B, Level III) [14,15]. At RIVM, cases by default had acute HCV infections, with either confirmed HIV-positive or unknown HIV-serostatus (captured since 2013). We determined the timing of HCV infection based on the reported date of laboratory diagnosis; if unavailable, the first date of illness, or the date of reporting to the RIVM was used.

### Case linkage and statistical analysis

We initially described the annual number of reported cases by HCV/HIV transmission risk group and HIV serostatus for the period 2003–2016, using descriptive statistics.

Subsequently, to facilitate capture–recapture analysis, cases of acute HCV infection were linked between the two databases for the period of 2013–2016. Cases were linked using a stepwise approach, based on: year and country of birth, sex, postal code (2 or 4 digits), municipal health service region, and date of HCV diagnosis (allowing a diagnostic timeframe). People who were living outside the Netherlands but who had been diagnosed and/or were in care in the Netherlands could not be matched on postal code and were matched based on municipal health service region. First, we linked the cases of acute HCV infection among cases with an HIV-positive serostatus. Second, we repeated the linkage and included additional cases of acute HCV infection reported to the RIVM with both a confirmed positive or unknown HIV serostatus.

Additionally, we assessed the influence of the distinction between acute and chronic HCV diagnosis on the registration in both databases. We linked RIVM cases of acute HCV infection among HIV-positive adults to all SHM cases of HCV infection (i.e. acute, chronic, and other). Subsequently, we repeated this analysis containing all RIVM cases of acute HCV infection, including cases with an unknown HIV serostatus.

#### The capture–recapture method

**Figure 1:**
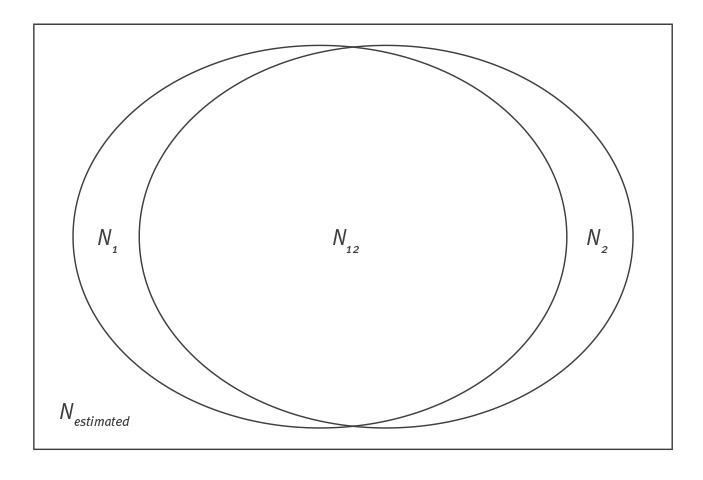
Venn diagram of capture–recapture analysis

HCV: hepatitis C virus; HIV: human immunodeficiency virus; *N_1_*: number of cases of HCV infections among adults with HIV registered by SHM; *N_2_*: number of cases of acute HCV infection among adults with HIV registered by RIVM^a^ *; N_12_*: number of cases of acute HCV infection among adults with HIV registered in common in SHM and RIVM databases; *N_estimated_*: estimated number of cases of acute HCV infection among adults with HIV; RIVM: National Institute for Public Health and the Environment; SHM: Dutch HIV Monitoring Foundation.

^a^ Cases of acute HCV among adults with an unknown HIV serostatus as registered by RIVM are added in a secondary analysis.

The capture–recapture method is derived from ecology and applied to epidemiological studies [18]. To estimate the number of cases in a population, cases are captured in one data source and then independently recaptured in a second data source. The capture–recapture method analyses the degree of overlap between the two data sources (Figure 1). The capture–recapture estimate is adequate when the following assumptions hold [19,20]: (i) the study applies to a closed population, i.e. the definition or interpretation of the ‘target population’ is explicit; (ii) linkage between the cases is possible and reliable; (iii) the databases are functionally independent and do not rely on each other; (iv) every case has the same probability to be included in each database.

For this study, we performed a two-way capture–recapture analysis to estimate the total number of cases of acute HCV among HIV-positive people based on the two databases for the period of 2013 to 2016. We calculated the adjusted Petersen–Lincoln estimate [19,20] of the total number of cases in 2013–2016, and by calendar year (i.e. 2013, 2014, 2015 and 2016) separately.

Adjusted Petersen–Lincoln estimated number of cases:

$$N_{estimated}=\frac{\left(N_{1}+1)(N_{2}+1\right)}{\left(N_{12}+1\right)}-1$$

With the associated variance of the estimate of the total population:

$$Variance=\frac{\left(N_{1}+1)(N_{2}+1)(N_{1}-N_{12})(N_{2}-N_{12}\right)}{\left(N_{12}-1{)}^{2}(N_{12}+2\right)}-1$$

And the 95% confidence interval (CI) of the estimate of the total population:

$$N_{estimated}\pm 1.96\sqrt{Variance)}$$

Where *N_1_* is the number of acute cases of HCV infections among adults with HIV registered by SHM, *N_2_* the number of cases of acute HCV infections among adults with HIV registered by RIVM, *N_12_* the number of cases of acute HCV infections among adults with HIV registered in common in SHM and RIVM databases and *N_estimated_* the estimated number of cases of acute HCV infection among adults with HIV.

### Ethical statement

At initiation, the ATHENA cohort [11] at SHM was approved by the institutional review board of all participating centres and patient consent is received by opting out. Data are pseudonymised (i.e. names and other personal identifiers are replaced by a reference number) before sharing with investigators and may be used for scientific purposes. The National Registry for Notifiable Diseases at RIVM anonymously registers mandatory communicable disease notifications [21], as a legal obligation enforced by the Dutch public health act [16] and International Health Regulations by the WHO [22].

For this study, we received approval from the steering committees of both institutions (i.e. RIVM and SHM). To secure non-traceability, SHM- and RIVM-specific reference numbers were removed from the datasets before case matching, using surrogate identifiers (i.e. new identifiers for the purpose of this study only, replacing the original RIVM and SHM reference numbers). A designated quality management coordinator safeguarded privacy protection and compliance with the European General Data Protection Regulation [23]. This work complies with the principles laid down in the Declaration of Helsinki [24].

## Results

### Acute hepatitis C virus infections among adults in 2003–2016

**Figure 2:**
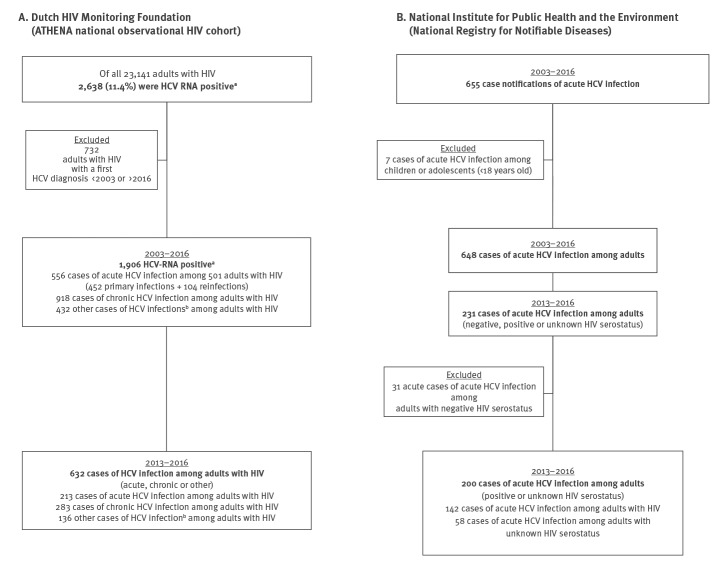
Extraction of cases of acute hepatitis C virus infections from the registries at the Dutch HIV Monitoring Foundation and the National Institute for Public Health and the Environment, the Netherlands, 2003–2016

HCV: hepatitis C virus; HIV: human immunodeficiency virus.

^a^ The number of HCV-RNA positive cases is equivalent to the number of cases of HCV infections because all seroconversions were confirmed with an HCV RNA positive test.

^b^ If the stage of HCV infection remained unresolved, cases of HCV infection were considered in a separate category called ‘other cases of HCV infection’ for the purpose of this study.

**Figure 3:**
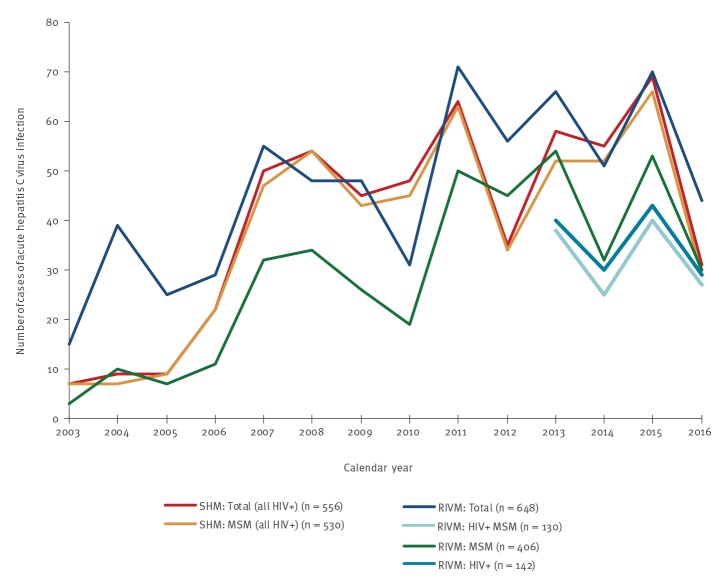
Cases of acute hepatitis C infection, as registered by the Dutch HIV Monitoring Foundation and the National Institute for Public Health and the Environment, the Netherlands, 2003–2016

HIV: human immunodeficiency virus; HIV+: HIV-positive case of acute hepatitis C infection; MSM: men who have sex with men; RIVM: National Institute for Public Health and the Environment; SHM: Dutch HIV Monitoring Foundation.

Cases of HCV infection were extracted from both registries as described in Figure 2, and summarised in Figure 3 and Supplemental Table 1.

For SHM, all cases of acute hepatitis C infections were among people with HIV. For RIVM, a positive HIV-serostatus is presented separately for men who have sex with men (MSM) only. In addition to the 130 cases among men with HIV who acquired HCV through sexual contact with other men (i.e. MSM), 12 cases of acute HCV infection were acquired through other sexual contact (n = 2, heterosexual and unknown), injecting drug use (n = 2), a needle stick- or bite-incident (n = 1), or other/unknown routes (n = 7). Also see Supplemental Table 1.

#### ATHENA cohort at the Dutch HIV Monitoring Foundation

In total, 2,638 (11.4%) of all 23,141 adults ever in HIV care and enrolled in ATHENA had a positive HCV-RNA test at least once (Figure 2). 

In the period of 2003–2016, 556 acute HCV infections were diagnosed among 501 HIV positive people: 452 were acute primary HCV infections and 104 were HCV reinfections. The 104 acute HCV reinfections were diagnosed among 86 people; the maximum number of acute HCV reinfections found per person was four (Supplementary Table 1). The number of people with an acute primary HCV infection is smaller than the total number of people with acute infections, because some people had a chronic HCV infection followed by an acute infection (a reinfection).

We restricted our capture–recapture analysis to the 213 cases of acute HCV infection among 205 HIV-positive adults in the period 2013–2016 (Figure 2). All but two cases were among males (n = 211; 99.1%), the median age at acute HCV diagnosis was 43 years (interquartile range (IQR): 36–50), and 171 (80.3%) were born in the Netherlands. Sexual contact among MSM was the main route of HIV infection (n = 199; 93.4%). The remaining 14 cases were in people who had acquired HIV through heterosexual contact (n = 6; 2.8%), injecting drug use (n = 2; 0.9%), receiving blood or blood products (n = 2; 0.9%), or another or unknown route (n = 4; 1.9%). Acute HCV was diagnosed a median 5.9 years (IQR: 2.6–10.7) after HIV diagnosis.

##### Cases of chronic hepatitis C virus infections and cases classified as other hepatitis C virus infections 

Additionally, 918 cases of chronic HCV infection and 432 other cases of HCV infection were registered in the period 2003–2016. Of 432 cases of other HCV infection, 357 people had a positive HCV antibody or RNA test followed by a negative HCV-RNA result, without having received HCV treatment. These people had likely spontaneously cleared the infection without treatment. Moreover, 75 of 432 people were diagnosed with HCV based on a single HCV-RNA test result without further diagnostic information. In the period 2013–2016, 283 cases of chronic HCV infection and 136 cases of other HCV infection were registered. The median time between HIV and subsequent HCV diagnosis was 5.7 years (IQR: 1.5–10.) and 5.6 years (IQR: 0.5–11.9) for cases of chronic HCV infection and cases of other HCV infection, respectively.

#### National Registry for Notifiable Diseases at the National Institute for Public Health and the Environment

In the period from 2003 to 2016, 655 cases of acute HCV infection were registered at the RIVM (**Figure 2**). Seven cases were excluded because they were among children or adolescents (< 18 years of age); five of seven had acquired HCV through vertical transmission. This left 648 cases of acute HCV infection among adults (Supplemental Table 1). The median age at diagnosis was 43 years (IQR: 35–49), and 89.0% were male (n = 577). Two-thirds of the cases were reported among people who were born in the Netherlands (n = 438; 67.6%). The main route of HCV transmission was sexual contact (n = 434; 67.0%), followed by injecting drug use (n = 50; 7.7%), or a needle stick or bite incident (n = 16; 2.5%); the remaining 148 infections were acquired through another or unknown transmission route (22.8%). Overall, 406 of all 648 (62.7%) acute HCV infections were acquired through sex between men (i.e. MSM).

There were 231 cases of acute HCV infection in 2013–2016 (Figure 2 **,** Table). Overall, 191 (82.7%) of 231 HCV infections were known primary infections and nine (3.9%) were reported as reinfections; data on primary/reinfection were unknown for the remaining 31 (13.4%) cases. In total, 142 of 231 (61.5%) cases were in HIV-positive men, 58 (25.1%) had an unknown or missing HIV serostatus (28 of whom were MSM), and 31 (13.4%) were HIV-negative (11 of whom were MSM). Of note, six of nine reported HCV reinfections occurred in HIV-positive people; for the remaining three reinfections, the HIV-serostatus was unknown. No reinfections were reported among HIV-negative people. 

**Table:** Number of cases of acute hepatitis C virus infection, by data source, after case-linkage, and combined estimates based on capture–recapture analysis, the Netherlands, 2013–2016

Year or period in years	Registered number of unique cases per database		Combined data			Capture–recapture analysis		% under-reporting SHM	% under-reporting RIVM
	SHM *N_1_*	RIVM *N_2_*	SHM only	RIVM only	RIVM-SHM *N_12_*	Total *N_estimated_* (95%CI)	Estimated unreported cases		
Cases with a positive HIV-serostatus									
2013	58	40	26	8	32	72 (65–80)	6	9.8	15.7
2014	55	30	33	8	22	74 (63–86)	11	17.3	38.0
2015	69	43	35	9	34	87 (78–96)	9	11.5	20.9
2016	31	29	12	10	19	47 (40–54)	6	16.2	20.6
2013–2016	213	142	106	35	107	282 (264–301)	34	13.9	19.5
Cases with both positive or unknown HIV-serostatus; also see Figure 3									
2013	58	60	16	18	42	83 (76–90)	7	10.4	11.1
2014	55	42	30	17	25	92 (76–108)	20	26.3	46.4
2015	69	59	29	19	40	101 (90–113)	13	16.3	22.7
2016	31	39	9	17	22	55 (47–62)	7	17.7	17.0
2013–2016	213	200	84	71	129	330 (309–351)	46	17.7	18.7

CI: confidence interval; *N_1_*: number of cases registered by SHM; *N_2_*: number of cases registered by RIVM; *N_12_*: number of cases registered in common in SHM and RIVM databases; *N_estimated_*: estimated number of cases; RIVM: National Institute for Public Health and the Environment (Rijksinstituut voor Volksgezondheid en Milieu); SHM: Dutch HIV Monitoring Foundation.

The capture–recapture estimates are calculated separately based on matched and non-matched cases, for each calendar year (2013, 2014, 2015, and 2016) and by the full period (2013–2016). Therefore, the estimate of the full period (2013–2016) does not equal the sum of the annual estimates.

Exclusion of the 31 cases of HCV infection with an HIV seronegative status resulted in 200 cases of acute HCV infection among adults with a positive (n=142) or unknown (n=58) HIV-serostatus who were used in the capture–recapture analysis. Among these, all of the 142 HIV-positive people were male, the median age at HCV diagnosis was 43 years (IQR: 35–51), and 118 (83.1%) were born in the Netherlands. Sexual contact among MSM was the main route of HCV infection (n = 130; 91.6%). The remaining 12 HCV infections were acquired through other sexual contact (n = 2, heterosexual and unknown), injecting drug use (n = 2), a needle stick or bite incident (n = 1), or another or unknown route (n = 7).

### Capture–recapture analysis for 2013–2016

**Figure 4:**
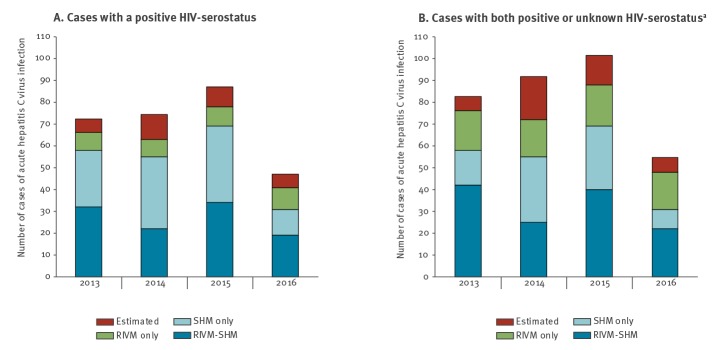
Capture–recapture estimates of cases of acute hepatitis C infections among adults with HIV in the Netherlands, 2013–2016

HIV: human immunodeficiency virus; RIVM: National Institute for Public Health and the Environment; SHM: Dutch HIV Monitoring Foundation.

^a^ Also see Table.

First, we linked the 213 and 142 cases of acute HCV infection among HIV-positive adults as registered in 2013–2016 by SHM and RIVM, respectively (Table). In total, 107 cases were found in both registries: 50.2% of the 213 SHM cases could be linked to RIVM cases, and 75.4% of the 142 RIVM cases to SHM cases. The median time between diagnosis as documented by SHM and RIVM was 56 days (IQR: 14–309); 74 (69.2%) cases were documented by SHM first, 29 (27.1%) were documented by RIVM first, and four (3.7%) were diagnosed on the same date. Based on capture–recapture analysis, a total of 282 (95%CI: 264–301) acute HCV infections among HIV-positive adults were estimated for the period of 2013–2016. The estimated annual number of cases was 72 (95%CI: 65–80) in 2013, 74 (95%CI: 63–86) in 2014, 87 (95%CI: 78–96) in 2015 and 47 (95%CI: 40–54) in 2016 (Figure 4A). Based on these estimates, under-reporting of acute HCV among HIV-positive people was 13.9% for SHM and 19.5% for RIVM.

Second, the addition of 58 RIVM-cases with an unknown HIV-serostatus led to an increase in the number of matches (Table). In total, 129 cases were found in both registries: 60.6% of the 213 SHM-cases, and 64.5% of the 200 RIVM cases. The median time between diagnosis as documented by SHM and RIVM was 56 days (IQR: 14–341); 90 (69.8%) cases were documented by SHM first, 36 (27.9%) were documented by RIVM first, and three (2.3%) were diagnosed on the same date. The addition of cases with an unknown HIV serostatus led to an increase of the overall estimated total number of acute HCV infections among HIV-positive adults: from 282 (95%CI: 264–301) to 330 (95%CI: 309–351) estimated cases in the period of 2013–2016 (Figure 4B). With the addition of RIVM cases with an unknown HIV serostatus, under-reporting of acute HCV among HIV-positive people was 17.7% for SHM and 18.7% for RIVM.

### Chronic and unknown stage hepatitis C virus infections

Additionally, we linked all 632 cases of HCV infection among HIV positive adults registered by SHM (i.e. acute, chronic, and other), with the 142 cases of acute HCV infection adults who were HIV positive, registered by the RIVM (Supplementary Table 2). All but two RIVM-cases could be linked to an acute, chronic, or other HCV case registered by SHM (98.6%; 140 of 142). Furthermore, when we added the 58 RIVM-cases of acute HCV infection among people with an unknown HIV serostatus -- i.e. when we linked the 632 SHM cases with the 200 RIVM cases -- we could link 175 cases of HCV infection, indicating under-reporting of HIV-positivity at RIVM and missed acute cases in the SHM data.

## Discussion

From 2003 to 2016, the predominant route of HCV transmission in the Netherlands, was sexual transmission among (HIV-positive) MSM. Based on data from the two national registries (SHM and RIVM), we estimated 282–330 cases of acute HCV infection among HIV-positive adults in the Netherlands, for the period of 2013–2016. Under-reporting was estimated at 14–18% for SHM and 19–20% for RIVM, and could partially be explained by an unknown HIV-serostatus at RIVM, or by differences between RIVM and SHM in HCV stage at first diagnosis (acute or chronic).

The results should be interpreted while taking into account the study's limitations. Notably, capture–recapture analysis is based on four assumptions, which influence the accuracy and reliability of the presented estimates [19,20]. Our study fulfils all these assumptions. However, incorrect case linkage and non-linkage cannot be ruled out because linkage was based on personal characteristics, and not on personal identifiers. Furthermore, while the SHM registry provided the possible route of HIV transmission, data on how HCV infection may have been acquired were only available in the RIVM registry. Therefore, the predominant HCV transmission route for MSM concluded in this report is based on the assumption that the risks profile to acquire both HIV and HCV were similar in such men registered in either database.

Our annual estimates of the number of acute HCV infections among people with HIV are in line with HCV incidence studies performed among HIV-positive MSM both in Amsterdam [25] and across Europe [26]. The estimated number of acute HCV infections was lower in 2016 than in 2013, 2014 and 2015, which could be explained by a delay in registration, a change in the frequency of HCV testing [27], or an actual decrease in HCV incidence among HIV-positive people in the Netherlands, most likely due to increased DAA uptake. The latter is in line with recently published findings covering 17 of 26 Dutch HIV-treatment centres, relating the high DAA uptake to a lower HCV incidence among HIV-positive MSM in 2016, than in 2014 [8]. However, annual numbers are known to fluctuate over time, also before 2013, and recent numbers reported by the RIVM showed a subsequent increase in the total number of HCV infections for 2017 and 2018 [7,28], compared with 2016.

Under-reporting at both registries could be explained by several factors. At SHM, under-reporting of acute HCV infections could be due to missed or incomplete screening for HCV by the HIV treating physician. Virtually all RIVM cases of acute HCV infection among adults with a known HIV-positive status could be identified at SHM, although some RIVM cases of acute HCV were registered as chronic HCV at SHM. Previous analyses of the SHM data have shown substantial variation in routine HCV screening for all HIV-positive people at entry into HIV care, as well as in annual HCV screening among risk groups (i.e. MSM) [5]. This may, to some extent, be explained by centres applying a policy of targeted screening guided by the presence of incident transaminase elevations [29]. Furthermore, complementary qualitative investigation has identified additional factors (guideline communication/presentation, patient, physician, and environmental factors) influencing the compliance to guidelines for laboratory monitoring in outpatient HIV care in the Netherlands [30]. At the RIVM, cases are reported through the local GGD, which receives case notifications from both the diagnostic laboratories and physicians in or outside of HIV care. A marked reduction in HCV screening at some Dutch sexually transmitted infection (STI) clinics among HIV-positive MSM could explain missed diagnoses and, as such, under-reporting of acute HCV infections, specifically among HIV-positive people not engaged in HIV care or not screened for HCV in HIV care. This is largely an effect of a change in screening policy by the Amsterdam STI clinic, the largest in the Netherlands, which had to discontinue HCV screening due to financial constraints in 2014; although HCV screening in HIV-positive MSM was restarted in 2017. However, the acute HCV infections among HIV positive people should have been diagnosed in HIV care and reported to the RIVM. Additionally, a large proportion of RIVM cases had a missing or unknown HIV-serostatus. Unknown HIV serostatus could occur when acute HCV is detected and notified by the laboratory (that is unaware of HIV-serostatus), or could be due to lack of HIV testing among HCV-positive people. The relatively high HCV prevalence observed among (HIV-negative) MSM starting pre-exposure prophylaxis (PrEP) in Europe [31-34] underscores the need for routine combined STI/HCV screening among MSM at high risk for HIV infection.

The sensitivity of surveillance systems relies on the likelihood of the infections being identified, diagnosed, and reported. We therefore recommend that all HCV infections, both acute and chronic, are registered at the National Registry for Notifiable Diseases at the RIVM, as in practice the timing of diagnosis and reporting by different healthcare workers can differ. Limiting the notification and registration to acute HCV infections only was primarily motivated by the need to trace sources and contacts. However, both the recent Dutch national action plan to eliminate hepatitis, and similar international hepatitis elimination aims, require comprehensive detection and registration of all HCV infections [1,3].

In order to achieve the European and global targets for hepatitis elimination, robust surveillance and monitoring data of viral hepatitis are needed to monitor progress. In the absence of national registries for viral hepatitis, linkage studies of other registries (than a national viral hepatitis registry) with relevant information can help countries to get insight in the completeness of the data in these respective registries, and can provide surveillance estimates. Our study confirmed that linkage of two national databases is possible, and can provide reliable surveillance data with additional value. Previously, van Leth et al. linked the national tuberculosis (TB) registry to SHM to assess the TB-HIV prevalence in the Netherlands [35]. In Denmark, linking multiple national registries has helped to estimate HBV and HCV prevalence [36,37]. Data linkage studies are versatile and can strengthen multi-disease surveillance and monitoring.

Infectious disease surveillance data are essential for the planning and evaluation of public health activities. To control and eventually eliminate HCV from the HIV-positive population in the Netherlands and beyond, all HIV/HCV co-infected people should be diagnosed, linked to, and retained in care [1,3]. Registration is essential to monitor trends in the HCV–HIV syndemic, and to assess the impact of treatment and other interventions on HCV incidence, both on a national and international level.

The relatively high HCV reinfection rates are a major obstacle to control the HCV epidemic, and to achieve HCV elimination targets. The high rate of HCV reinfections among HIV-positive MSM [5,38] warrants the need for increased awareness and prevention measures for high risk MSM, as well as structural repeat screening of HCV infection [39,40]. There is a need for more data on reinfection rates in the DAA era (as opposed to data on reinfection rates dating from the era when interferon-based treatment was used), which both describe the rates, as well as the associated factors, in order to develop targeted interventions to reduce HCV reinfection [41]. Among HIV-positive MSM, both HCV treatment and behavioural risk reduction are essential to move towards HCV elimination in this population [42,43]. We call for increased attention for both documentation of HCV cases in medical records and disease notification at RIVM, by all health professionals. Surveillance data should ideally include both acute and chronic HCV infections, and be able to distinguish between these, as well as primary infections and reinfections. Robust HCV surveillance is essential to monitor the impact of prevention programmes and DAA treatment uptake – on both the individual and public health level.

## Data statement

The data used in this study are confidential and would not be shared. The ATHENA cohort data are available on request at SHM; for more information: https://www.hiv-monitoring.nl/english/research/research-projects/research-proposal/.

## *Authors’ correction

At the request of the authors, in the conflict of interest section, the sentence regarding the conflict of interest of MvdV was amended. The changes to this sentence were made on 21 February 2020. The sentence on MP’s institution was added on 26 February 2020 in the conflict of interest section.

## Supplementary Data

223000 Supplementary Material
