# Authors’ correction for Euro Surveill. 2020;25(7)

**DOI:** 10.2807/1560-7917.ES.2020.25.8.20200227c

**Published:** 2020-02-27

**Authors:** Eurosurveillance editorial team

**Affiliations:** 1 European Centre for Disease Prevention and Control (ECDC), Stockholm, Sweden

In the article ‘Acute hepatitis C infection among adults with HIV in the Netherlands between 2003 and 2016: a capture–recapture analysis for the 2013 to 2016 period’ by Boender et al. published on 20 February 2020, at the request of the authors, two sentences in the conflict of interest section were amended. The changes were made on 21 and 26 February 2020.

