# Supplementary material for: Acute hepatitis C infection among adults with HIV in the Netherlands between 2003 and 2016: a capture–recapture analysis for the 2013 to 2016 period
Source: Euro Surveill. 2020 Feb 20;25(7):1900450. doi: 10.2807/1560-7917.ES.2020.25.7.1900450 (PMC7043050; doi:10.2807/1560-7917.ES.2020.25.7.1900450)
Supplement: Supplementary Material [file 1900450_BOENDER_Supplementary_Material.pdf]

Supplemental Tables for manuscript: "Acute hepatitis C infection among adults with HIV in the Netherlands between 2003 and 2016: a capture–-recapture analysis for the 2013 to 2016 period"

This supplementary material is hosted by *Eurosurveillance* as supporting information alongside the article "Acute hepatitis C infection among adults with HIV in the Netherlands between 2003 and 2016: a capture–-recapture analysis for the 2013 to 2016 period", on behalf of the authors, who remain responsible for the accuracy and appropriateness of the content. The same standards for ethics, copyright, attributions and permissions as for the article apply. Supplements are not edited by *Eurosurveillance* and the journal is not responsible for the maintenance of any links or email addresses provided therein.

Supplemental Table 1. Acute hepatitis C virus infection cases, per registration database, by transmission route and HIV-serostatus

Year	RIVM				SHM	
	Total	MSM	HIV-positive	MSM HIV-positive	HIV-positive	MSM HIV-positive
	N	N	N	N	N	N
2003	15	3			7	7
2004	39	10			9	7
2005	25	7			9	9
2006	29	11			22	22
2007	55	32			50	47
2008	48	34			54	54
2009	48	26			45	43
2010	31	19			48	45
2011	71	50			64	63
2012	56	45			35	34
2013	66	54	40	38	58	52
2014	51	32	30	25	55	52
2015	70	53	43	40	69	66
2016	44	30	29	27	31	29
total	648	406	142	130	556	530

MSM=men who have sex with men; determined based on HCV transmission route for RIVM and HIV transmission route for SHM.

Supplemental Table 2. Number of confirmed acute hepatitis C virus (HCV) infections: by data source, after case-linkage

Based on A) All HCV cases (acute, chronic, other) with a positive HIV-serostatus; and

B) HCV cases (acute, chronic, other) with both positive or unknown HIV-serostatus.

	**Registered number of unique cases per database**		**Combined data**		
**A) All HCV positive cases with a positive HIV-serostatus**					
Year	SHM	RIVM	SHM only	RIVM only	SHM-RIVM
	N	N	N (N_1)	N (N_2)	N (N_{12})
2013	167	40	127	0	40
2014	174	30	145	1	29
2015	171	43	128	0	43
2016	120	29	92	1	28
2013-2016	632	142	492	2	140
**B) All HCV positive cases with both positive or unknown HIV-serostatus.**					
Year	SHM	RIVM	SHM only	RIVM only	SHM-RIVM
	N	N	N (N_1)	N (N_2)	N (N_{12})
2013	167	60	112	5	55
2014	174	42	138	6	36
2015	171	59	117	5	54
2016	120	39	90	9	30
2013-2016	632	200	457	25	175
